# Bovine Milk-Derived Emulsifiers Increase Triglyceride Absorption in Newborn Formula-Fed Pigs

**DOI:** 10.3390/nu13020410

**Published:** 2021-01-28

**Authors:** Kristine Bach Korsholm Knudsen, Christine Heerup, Tine Røngaard Stange Jensen, Xiaolu Geng, Nikolaj Drachmann, Pernille Nordby, Palle Bekker Jeppesen, Inge Ifaoui, Anette Müllertz, Per Torp Sangild, Marie Stampe Ostenfeld, Thomas Thymann

**Affiliations:** 1 Department of Veterinary and Animal Science, University of Copenhagen, 68 Dyrlægevej, DK-1870 Frederiksberg, Denmark; kristine.bach.korsholm.knudsen@regionh.dk (K.B.K.K.); tine.stange@hotmail.com (T.R.S.J.); pts@sund.ku.dk (P.T.S.); 2 Rigshospitalet, Department of Pediatric Surgery, 9 Blegdamsvej, DK-2100 Copenhagen, Denmark; inge.ifaoui@regionh.dk; 3 Department of Pharmacy, University of Copenhagen, 2 Universitetsparken, DK-2100 Copenhagen, Denmark; christine.heerup@sund.ku.dk (C.H.); anette.mullertz@sund.ku.dk (A.M.); 4 Department of Food Science, University of Copenhagen, 26 Rolighedsvej, DK-1958 Frederiksberg, Denmark; xiage@arlafoods.com; 5 Arla Foods Ingredients, 10-12 Soenderhoej, DK-8260 Viby, Denmark; nidra@arlafoods.com (N.D.); mstos@arlafoods.com (M.S.O.); 6 DTU Bioengineering, Technical University of DK, Søltofts Plads, DK-2800 Kongens Lyngby, Denmark; nordby.p@gmail.com; 7 Rigshospitalet, Department of Gastroenterology, 9 Blegdamsvej, DK-2100 Copenhagen, Denmark; Palle.Bekker.Jeppesen@regionh.dk

**Keywords:** preterm neonates, fat, gastric lipase, absorption, intestine, milk, emulsions, vegetable oil, soy lecithin

## Abstract

Efficient lipid digestion in formula-fed infants is required to ensure the availability of fatty acids for normal organ development. Previous studies suggest that the efficiency of lipid digestion may depend on whether lipids are emulsified with soy lecithin or fractions derived from bovine milk. This study, therefore, aimed to determine whether emulsification with bovine milk-derived emulsifiers or soy lecithin (SL) influenced lipid digestion in vitro and in vivo. Lipid digestibility was determined in vitro in oil-in-water emulsions using four different milk-derived emulsifiers or SL, and the ultrastructural appearance of the emulsions was assessed using electron microscopy. Subsequently, selected emulsions were added to a base diet and fed to preterm neonatal piglets. Initially, preterm pigs equipped with an ileostomy were fed experimental formulas for seven days and stoma output was collected quantitatively. Next, lipid absorption kinetics was studied in preterm pigs given pure emulsions. Finally, complete formulas with different emulsions were fed for four days, and the post-bolus plasma triglyceride level was determined. Milk-derived emulsifiers (containing protein and phospholipids from milk fat globule membranes and extracellular vesicles) showed increased effects on fat digestion compared to SL in an in vitro digestion model. Further, milk-derived emulsifiers significantly increased the digestion of triglyceride in the preterm piglet model compared with SL. Ultra-structural images indicated a more regular and smooth surface of fat droplets emulsified with milk-derived emulsifiers relative to SL. We conclude that, relative to SL, milk-derived emulsifiers lead to a different surface ultrastructure on the lipid droplets, and increase lipid digestion.

## 1. Introduction

Exclusive breastfeeding is, according to WHO, the preferred nutrition from birth to the age of six months [1], yet worldwide this is only accomplished for approximately 41% of all infants [2]. Reasons for this include mothers who are unable or chose not to breastfeed or have complications such as preterm birth [3]. Whereas donor milk may be an alternative, particularly for preterm infants [4], high-quality formulas are required when donor milk is not available to ensure survival and normal development. Relative to breastfed infants, formula-fed infants have an increased risk of developing atopic diseases [5], respiratory infections [6], necrotizing enterocolitis [7], and other gastrointestinal complications [8]. There is also evidence of a higher risk of reduced neurodevelopment in formula-fed infants than in infants fed mothers’ milk, even after adjusting for important confounders [9,10]. These effects on cognitive outcomes have been observed in both preterm and term infants [9], where formula-fed infants have lower IQ and a lower score for cognitive functions [10,11], which may persist into later life [12,13].

Lipid supplementation to the brain is essential for normal neurological development. Accumulation of lipids in the brain begins in the third trimester and continues the first two years of postnatal life [14]. Especially long-chain polyunsaturated fatty acids (LC-PUFAs) are important, as they represent essential cell membrane components in the brain. A lower concentration of LC-PUFAs in the brain has been observed in formula-fed infants and they also have poorer neurological outcomes [15,16,17]. There are differences between breast milk and infant formula with regard to the bioavailability of LC-PUFAs, which may partly explain the differences observed in neurodevelopment [18]. However, compensating for lower bioavailability by supplementing infant formula with more LC-PUFAs has not shown any cognitive improvement [19,20].

One reason for the lack of improved neurological outcomes from formulas high in LC-PUFA may be low intestinal absorption leading to low delivery of LC-PUFA to the brain. Fat absorption in infants is generally less efficient relative to adults, and this is even more pronounced in preterm infants who have an absorption rate of 70–80% relative to 95% in adults. This is mainly due to the involvement of different lipases in infants than in adults and thereby different digestive capacity [21]. Moreover, formula-fed infants have reduced fat digestion and absorption relative to infants fed mother’s milk [22,23]. Accordingly, lipid digestion in formula-fed infants should be improved to approximate breastfed infants’ absorption levels. In mother´s milk, lipids in the form of triglycerides, are mainly transported as milk fat globules surrounded by a milk fat globule membrane (MFGM) [24]. Milk fat globules are secreted into the milk from the mammary gland by a unique mechanism giving MFGM a triple-layered membrane. This is a highly complex membrane containing several classes of phospholipids (including sphingomyelin), and glycosphingolipids, cholesterol, and unique membrane proteins, many of which are highly glycosylated [25]. MFGM forms a hydrophilic layer around the triglyceride core, making the fat globules water-soluble in the milk’s water matrix [26,27]. Another phospholipid-rich source in milk is extracellular vesicles (EVs) [28]. EVs are also secreted from the mammary gland and consist of a lipid bilayer membrane comprised of phospholipids, glycerosphingolipids, cholesterol, and membrane proteins, but are devoid of a central triglyceride core. The EV membrane is rich in sphingomyelin and cholesterol as well as tetraspanins CD9, CD63, and CD81.

In infant formula today, soy lecithin (SL) is commonly used as an emulsifier and stabilizer. SL mainly consists of phosphatidylcholine and does not contain sphingomyelin or cholesterol, both of which play a major role in forming the lipid rafts found in MFGM [27]. Thus SL provides a different surface structure on the lipid droplets in infant formula [29]. Moreover, human milk lipids are more readily digested in preterm infants compared with infant formula [23]. Lipid digestion in infants has been examined using an in vitro model, simulating infant gastrointestinal conditions. This model used human gastric aspirate as a source of gastric lipase and porcine pancreatin as a source of pancreatic lipase. A higher in vitro gastric lipolysis rate was found when emulsifying lipids with milk phospholipids relative to SL, and this was also the case for intestinal lipolysis rate [30].

On this background, we hypothesized that emulsification with bovine phospholipid sources such as whey protein concentrate enriched in phospholipids (WPC-PL) or WPC from acid whey-enriched in extracellular vesicles (WPC-A-EV) with their unique composition of glycolipids, phospholipids, sphingomyelin, and glycosylated proteins joined in a complex membrane structure, would enhance digestion of dietary lipids compared to SL. Accordingly, the objective was to emulsify vegetable-based oils with either SL or WPC-PL or WPC-A-EV and determine their effect on the in vitro rate of lipolysis, and in vivo digestion and absorption of triglycerides. To determine any influence of emulsifiers on lipid digestion, we chose to use a preterm neonatal piglet model. This was from the assumption that prematurity per se would associate with lower digestive capacity, thereby making any potential improvements of lipid digestion more clear.

## 2. Materials and Methods

### 2.1. In Vitro Lipolysis

For the in vitro lipolysis studies, five oil-in-water emulsions were made using either SL (AAK, Karlshamn, Sweden), or bovine whey protein concentrate enriched in phospholipids (WPC-PL), whey protein concentrate from acid whey enriched in triglycerides (WPC-A-TAG), whey protein concentrate from acid whey enriched in EVs (WPC-A-EV), or whey protein concentrate from acid whey enriched in soluble whey protein (WPC-A-WP). All bovine products were kindly donated by Arla Foods Ingredients Group P/S, and the composition of the emulsifiers is provided in Table 1.

The emulsions were prepared using the emulsification method, composition, and oil-blend described in Heerup et al. (submitted) [31]. In brief, the emulsions were made with 0.35% emulsifier and 3.5% oil-blend (98.92% Akonino NS (AAK, Karlshamn, Sweden), 0.97% MEG-3 (DSM, Mulgrave, NS, Canada), and 0.51% Arasco (DSM)) in an aqueous 11.5 mM CaCl_2_ and 8.5 mM NaCl solution.

The emulsions were digested using the in vitro pediatric gastro-intestinal digestion model described in Heerup et al. In brief, the model consisted of a 50 min gastric step at pH 6.4 with 3.75 TBU/mL recombinant human gastric lipase (rHGL) kindly donated by Bioneer A/S (Hørsholm, Denmark) and 126 U/mL pepsin purchased from Sigma Aldrich (St. Louis, MO, USA), a 90 min intestinal step at pH 6.5 with 26.5 TBU/mL pancreatin (Sigma Aldrich), and a back titration to pH 9. Since the SL emulsion was not stable in the 11.5 mM CaCl_2_ and 8.5 mM NaCl solution, CaCl_2_ and NaCl were instead added as part of the gastric medium. Table 2 shows the final gastric and intestinal assay composition, including the contribution of CaCl_2_ and NaCl from the emulsions. The degree of lipolysis over time was measured indirectly by continuous titration of ionized free fatty acids with 0.2 mM NaOH using a Metrohm Titrando pH Stat (Metrohm, Glostrup, Denmark). The particle size distribution of the undigested emulsions was measured on the day of lipolysis.

### 2.2. Microstructure of Emulsions

To determine structural differences we selected a subfraction of emulsions, i.e., SL, WPC-PL, and WPC-A-EV. These emulsions were visualized with transmission electron microscopy (TEM) and cryo-scanning electron microscopy (SEM). The emulsions used for TEM and SEM were prepared according to the method described by Heerup et al., although with no CaCl_2_ or NaCl added to any of the emulsions. The emulsions were mixed 1:1 with 2% agarose (Carl Roth, Germany) at 37 °C and left at room temperature for solidification. Several small pieces of the solidified sample were cut and fixed in the 2% glutaraldehyde phosphate buffer (pH 7.2) for 30 min at room temperature, followed by washing and postfix in 1% *w/v* OsO_4_ with 0.05 M K_3_Fe(CN)_6_ in 0.12 M phosphate buffer (pH 7.2) for 2 h. After that, a standard procedure for dehydration, embedding, and sectioning was applied. Finally, the ultra-thin sectioned sample (~60 nm) were collected on copper grids with Formvar supporting membranes, stained with uranyl acetate and lead citrate, and examined by a Philips CM-100 electron microscope (Philips, Eindhoven, The Netherlands) operated at 100 kV. For SEM, specimens were sandwiched in 2 × 100 µm planchettes and cryopreserved by high-pressure freezing (HPM100, Leica, Vienna, Austria). The sandwiched planchettes were mounted in a planchette holder (Leica) under liquid N_2_ and transferred to a vitreous cryo transfer shuttle (VCT100, Leica). The samples were cracked and sputter-coated (approximately 6 nm) (MED020, Leica) with carbon/platinum. Specimens were examined with an FEI Quanta 3D scanning electron microscope operated at an accelerating 2 kV voltage.

### 2.3. Preparation of Emulsions for In Vivo Studies

From the initial five emulsifiers tested in the in vitro system, we selected WPC-PL and SL for in vivo study 1 and 2. While WPC-PL was chosen because it had shown a beneficial effect in infant formula [32], we chose SL as it is a common emulsifier and stabilizer often used in infant formulas. In the in vivo study, three were used as the most promising experimental emulsifier (WPC-A-EV) along with WPC-PL and SL to validate the improved digestibility observed in vitro. Ideally, all three emulsifiers could have been studied in each in vivo experiment but it was not feasible to include so many groups as the studies were very labor-intensive. To prepare the emulsions, we used a Rannie homogenizer (APV, Copenhagen, Denmark) at pressure 25 bar/250 bar, instead of a microfluidizer. The oil-in-water emulsions for the in vivo studies were made with 10% oil (*w*/*w*%) using an oil-blend containing 91.66% Akonino NS, 5.46% MEG-3, and 2.88% Arasco, and 1% emulsifier. Different experimental diets were used for each of three in vivo studies: In in vivo study 1, the experimental diet consisted of complete formulas based on 10% oil-in-water emulsions with either SL (VWR, Darmstadt, Germany) or WPC-PL mixed with a base-diet to achieve a final fat concentration of 5.1%. The base-diet was made from whey protein (Lacprodan^®^ DI-9224), casein (Miprodan^®^ 40), lactose, and minerals (Variolac^®^ 855, all Arla Foods Ingredients) and was designed to meet the nutritional needs of pigs. In in vivo study 2, the experimental diet consisted of pure 10% oil-in-water emulsions with either SL (VWR) or WPC-PL. Finally, in in vivo study 3, the experimental diet consisted of complete formulas based on 10% oil-in-water emulsions with either SL (AAK, Aarhus, Denmark), WPC-PL, or WPC-A-EV mixed with the base diet to a final fat concentration of 10%.

### 2.4. In Vivo Lipid Digestibility of Complete Formulas (Study 1)

All procedures were approved by the Danish Animal Experiments Inspectorate (license number 2014-15-0201-00418), which follows the guidelines from Directive 2010/63/EU of the European Parliament and the ARRIVE guidelines [33]. We used cesarean-derived preterm neonatal piglets as they were assumed to have lower fat digestive capacity relative to term pigs, thereby making any effect of emulsifiers more detectable. In brief, one litter of preterm piglets, *n* = 22, (Landrace × Large white × Duroc, Gadstrup, Denmark) was born by cesarean section at day 113 of gestation and reared in preheated and oxygenated incubators as described previously [34]. Immediately after birth, the pigs were equipped with oral and vascular catheters to allow enteral and parenteral feeding. See supplemental for further information. On the second day, the pigs were surgically equipped with a jejunostomy to allow the quantitative collection of stoma output. Details for housing, feeding, surgery, post-surgical care, and sample collection are provided in the supplemental material. The piglets were block-randomized according to bodyweight into two groups receiving complete formulas emulsified with either SL (*n* = 5–9) or WPC-PL (*n* = 6–9). Enteral feeding was initiated as quickly as possible postoperatively at a rate of 6 mL/kg every three hours, gradually increasing to 15 mL/kg every three hours on day seven and eight. The personnel were blinded to the treatment groups. Stoma output was collected quantitatively on days 3, 4, and 7, and following measurement of fat concentration in the stoma output, intestinal fat absorption was calculated as described earlier [35].

Fat accumulation in the tissues of the small intestine was measured. A piece of the proximal part of the small intestine was fixed in a cryo cassette (Tissue-Tek Cryomold, Sakura Finetek, Zoeterwouder, Holland, The Netherlands) with tissue O.C.T. (Tissue-Tek, Sakura, Finetek Zoeterwoude, Holland) and frozen in liquid nitrogen. The tissue was sliced with a cryostat (Leica CM 1950, Leica Biosystems, Wetzlar Germany), and stained with Oil-Red-O (Sigma-Aldrich, Darmstadt, Germany). Six digital images were taken at 20× magnification from six different regions using a microscope (Olympus BX45, Tokyo, Japan), and the degree of fat infiltration was scored.

### 2.5. In Vivo Fat Absorption Kinetics of Pure Emulsions (Study 2)

Subsequently, we examined the triglyceride absorption kinetics after dosing of pure 10% oil-in-water emulsions. One litter of preterm piglets, *n* = 19, was born by cesarean section at day 106 of gestation, making the piglets 7 days more premature relative to the first study which further sensitizes the gut toward low digestive capacity. We have used this degree of prematurity in many previous experiments and based on sensitivity to develop prematurity-related complications like necrotizing enterocolitis, which may correspond to week 25–28 in human pregnancy. Procedures for cesarean section, postnatal rearing, and provision of parenteral nutrition were identical to the previous study. On day two, the piglets were blocked randomized according to body weight to receive an enteral bolus (6 mL/kg body weight) of pure emulsion with either SL or WPC-PL. Using a cross-over design, the piglets received a second pure emulsion bolus on day three, such that each piglet had been exposed to both emulsions. Blood samples from the arterial umbilical catheter were collected on both days at t = 0, 90, 180, 270, 360, and 540 min after the bolus was given and stored in heparinized tubes. Plasma was isolated after centrifugation (1270× *g*, 4 °C, 10 min), and the concentration of triglycerides was analyzed using an automated ADVIA 1800 Chemistry System (Siemens Healthcare A/S, Ballerup, Denmark). Following blood sampling, all pigs were euthanized.

### 2.6. In Vivo Lipid Absorption Kinetics from Complete Formulas (Study 3)

In a final in vivo experiment, the triglyceride absorption kinetics following ingestion of complete formulas was determined. A total of 49 preterm piglets from three pregnant sows were born by cesarean section at day 106 of gestation. They were stabilized and received parenteral nutrition as described above and were block-randomized according to birthweight into three groups, all receiving the complete formula, with a fat content of 10%. Collectively for all three in vivo studies, we ensured to have pigs from each litter equally represented in all treatment groups, allowing us to correct for any variance between litters and their specific genotype, in the analysis of variance. The complete formulas were made with the emulsions based on SL, WPC-PL, or WPC-A-EV. To stimulate the intestinal absorptive function, we initially fed the pure base-diet without emulsions during the first 24 h, at a rate of 3 mL/kg every three hours. On day two, the complete formulas including the emulsions were given at a bolus dose of 9 mL/kg, following a 6 hr fasting period. Blood samples were collected via the umbilical catheter at t = 0, 30, 60, 90 min after the bolus. Following this, the piglets were again fed with increasing amounts of enteral diet (6–9 mL/kg every three hours). On day four, the piglets were again fed a test meal of 9 mL/kg after a fasting period of 3.5 h, and blood samples were collected at t = 0, 30, 60, 90, and 120 min. Blood was drawn via the jugular vein in cases where the umbilical catheter was dysfunctional. Plasma was isolated, and triglyceride concentration was measured.

The pigs were euthanized on day four following a standardized feeding regimen to ensure an equal amount of gastric content at the time of euthanasia. Specifications for recordings and sample collection are provided in the supplementary section. Gastric content was weighed, and gastric lipase activity in gastric content was measured using the method described earlier with slight modifications [36]. The assay was carried out using the same pH Stat equipment as for the in vitro lipolysis. It was initiated by mixing 14.5 mL assay medium containing 1.5 µM Bovine Serum Albumin (AppliChem, Darmstadt, Germany), 150 mM NaCl (VWR), and 2 mM sodium taurodeoxycholate (Sigma Aldrich) with 0.5 mL tributyrin (Sigma Aldrich) and 0.5 mL stomach content. The enzymatic activity, calculated as U/mL was based on the measured titration rate of NaOH by a Metrohm Titrando pH Stat over five minutes of digestion at pH 5.5. As the butyric acid was not being fully titratable at pH 5.5, a correction factor of 1.12 was multiplied to the calculated activity. The total amount of collected stomach content, as well as the pH, was also measured.

## 3. Statistics

Statistical analyses for the in vitro digestions were performed using GraphPad Prism version 7.0 for Windows (GraphPad Software, San Diego, CA, USA, www.graphpad.com). Group comparisons were made using unpaired t-tests based on the area under the curve (AUC).

Statistical analyses for the in vivo studies were performed using R (version 3.5.0, R Foundation for Statistical Computing, Vienna, Austria). Continuous data were analyzed with a linear mixed model using the lm function (lme4 package). Group comparisons were made with an ANOVA (lme4 package) and Post Hoc Tukey test with the glht function (multcomp package). Normal distribution and homoscedasticity of residuals were visualized for model validation. Birthweight was used as a covariate, sex as a fixed variable, and litter as a random variable in all the models. Repeated blood samples were analyzed using SAS (SAS Software 9.4, SAS Institute, Cary, NC, USA). In all repeated measures models, birthweight and baseline triglyceride levels were included as covariates, whereas sex and litter were included as fixed and random effects respectively. Survival curves of basic motor skills were evaluated in GraphPad (Prism version 7.0) using the Logrank test for the trend. Fat accumulation scores were analyzed as ordinal data with the clmm function (ordinal package), and group comparison was made with the post hoc Tukey test. Group comparisons of the gastric lipase activity were made with unpaired t-tests in the GraphPad (Prism version 7.0). *p*-values < 0.05 were regarded significant and *p*-values < 0.1 as a tendency to effect. Data are presented as means ± standard deviation unless otherwise stated.

## 4. Results

### 4.1. In Vitro Lipolysis

**Figure 1:**
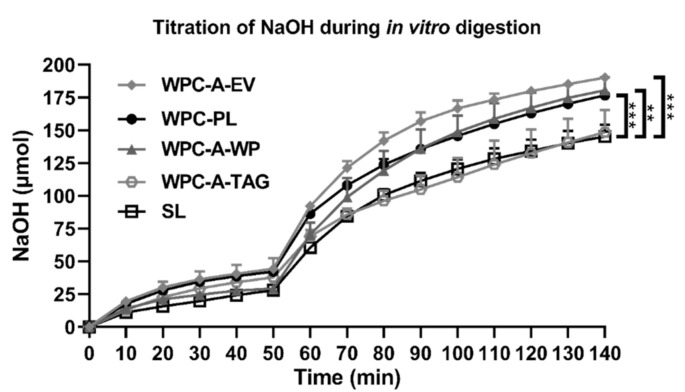
Titration of free fatty acids during simulated human gastric digestion with recombinant human gastric lipase (rHGL) and pepsin at pH 6.4 (0–50 min) and pH 6.5 (50–140 min) of 3.5% oil-in-water emulsions using either whey protein concentrate from acid whey-enriched in extracellular vesicles (WPC-A-EV) (*n* = 3), WPC enriched in phospholipids (WPC-PL) (*n* = 3), whey protein concentrate from acid whey enriched in triglycerides (WPC-A-TAG) (*n* = 3), whey protein concentrate from acid whey enriched in soluble whey protein (WPC-A-WP) (*n* = 3), and soy lecithin (SL) (*n* = 2 due to removal of outliers). Values are presented as mean ± SD. ** *p* < 0.001. *** *p* < 0.0001.

The in vitro lipolysis assessed as the amount of released free fatty acids (FFAs) titrated over time during digestion of each of the five 3.5% fat emulsions, using SL, WPC-PL, WPC-A-EV, WPC-A-TAG, and WPC-A-WP, is presented in Figure 1. After 50 min of in vitro digestion 28–44 µmol of FFAs were titrated depending on the emulsifier (SL: 28.1 µmol ± 0.5, WPC-A-WP: 29.3 µmol ± 1.6, WPC-A-TAG: 38.0 µmol ± 1.7, WPC-PL: 42.2 µmol ± 0.6, and WPC-A-EV: 44.3 µmol ± 6.6). After 140 min 145–190 µmol of FFAs were titrated (SL: 145.2 µmol ± 6.4, WPC-A-TAG: 148.6 µmol ± 13.7, WPC-PL: 176.8 µmol ± 1.1, WPC-A-WP: 180.6 µmol ± 8.0, and WPC-A-EV: 190.2 µmol ± 2.0). The in vitro digestion of emulsions based on WPC-A-EV, WPC-PL, and WPC-A-WP showed a higher lipolysis rate relative to SL. This was found, both when considering the AUC of the gastric and intestinal step separately, as well as the two combined (all *p* < 0.01), whereas WPC-A-TAG showed values similar to SL.

### 4.2. In Vitro Study 2—Microstructure of Emulsions

**Figure 2:**
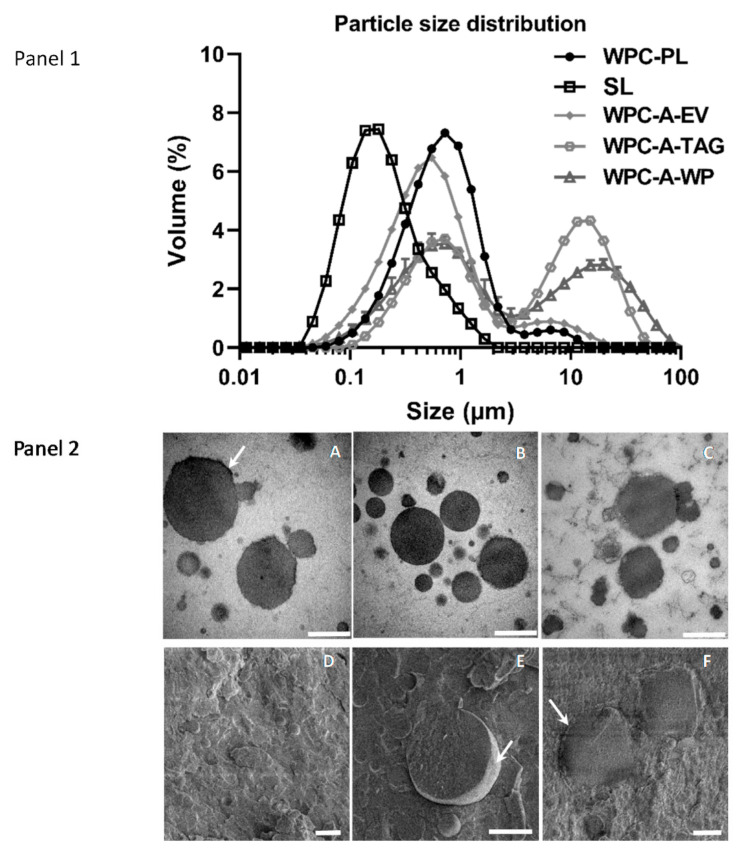
Particle size distribution and transmission electron microscopy (**Panel 1**): Volume mean diameter for each of the 3.5% oil-in-water emulsions, WPC-A-EV, WPC-PL, WPC-A-TAG, WPC-A-WP, and soy lecithin (SL). *n* = 3. Values are presented as means ± SD. (**Panel 2**): Micrographs from transmission electron microscopy (subpanels A, B, C) and cryo-scanning electron microscopy (subpanels D, E, F) of three emulsions. A and D: WPC-PL emulsion, B and E: WPC-A-EV emulsion, C and F: SL emulsion. The scale bar for micrographs A–C is 500 nm, and D–F is 2 µm. The arrow in A points out the dark layer, in E the arrow points out the lipid droplet’s smooth surface, and in F the arrow points out the thick and uneven surface of the lipid droplet.

The droplet size distribution for each of the five 3.5% fat emulsions, SL, WPC-PL, WPC-A-EV, WPC-A-TAG, and WPC-A-WP, are presented in Figure 2, Panel 1 as the volume mean diameter. All emulsions had droplet sizes within the range of 0.04–100 µm, with the smallest droplet size being observed for the SL emulsion, which had a unimodal distribution centered at 0.16 µm. WPC-A-EV and WPC-PL emulsions had similar droplet size distributions, and both had a primary population with modes centered at 0.5–0.7 µm and a smaller population around 7 µm.

The droplet sizes in the WPC-A-TAG and WPC-A-WP emulsions were similar and showed two distributions of nearly uniform height, with the first at 0.6 µm for both WPC-A-TAG and WPC-A-WP and the second mode around 4 µm for WPC-A-TAG and 17 µm for WPC-A-WP. The microstructure of WPC-PL, WPC-A-EV, and SL emulsion was further characterized by TEM (Figure 2, Panel 2). In general, the lipids droplets in the WPC-PL (subpanels A and D) and WPC-A-EV (subpanels B and E) emulsions had a similar structure with clear edges and relatively round and smooth appearances compared with droplets in the SL emulsion (subpanels C and F). Some of the droplets in the WPC-PL emulsions were partially covered by a thick dark layer (subpanel A), which might be composed of milk protein (mainly from aggregated whey proteins) from the WPC-PL product. These thick layers were less frequently observed for the droplet made of WPC-A-EV (subpanel B). Due to the higher content of native whey proteins present in WPC-A-EV relative to WPC-PL (communication with Arla Foods Ingredients), less aggregated protein complex can be associated with the interface of the lipid droplet presumably due to lower protein content in WPC-A-EV relative to WPC-PL. The SL lipid droplets had a different morphology than that of the other two emulsions, with irregular and uneven appearances (subpanel C). Besides the fluffy and thick layer of phospholipids on the droplet’s surface, we also observed a white layer of particles that either lies on the surface of the lipids droplets or are incorporated within the droplets. These droplets may be nano-sized liposomes generated during homogenization. Further, the cryo-SEM images provided a 3D cross-section view of the emulsions, which indicated that the surface of lipid droplets in the WPC-PL and WPC-A-EV emulsions were thinner and smoother relative to the SL emulsion droplets.

### 4.3. In Vivo Study 1—In Vivo Fat Digestibility of Complete Emulsions

**Figure 3:**
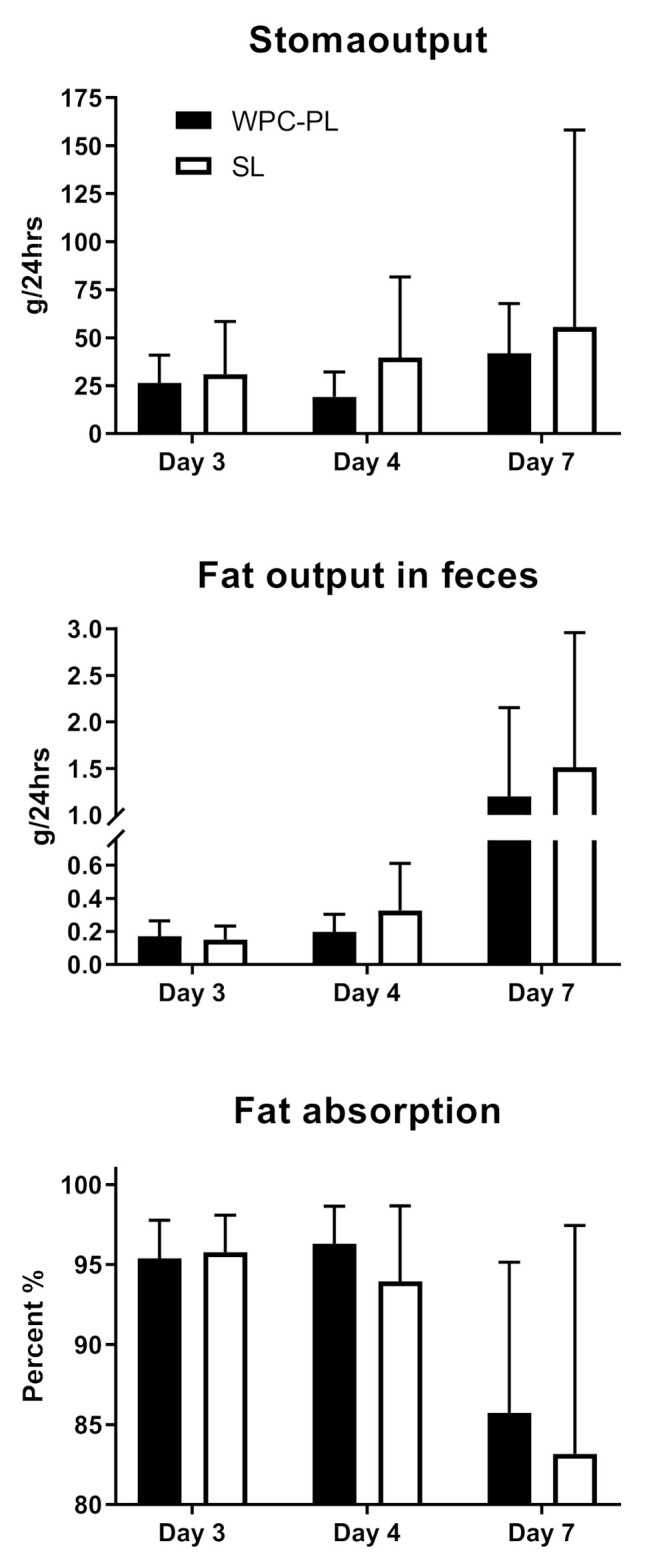
Total fecal stoma output (**upper panel**), total fat output in the feces (**middle panel**) and fat absorption percentages (**lower panel**) on day 3, 4, and 7 (WPC-PL *n* = 6–9, SL *n* = 5–9). Values presented as mean ± SD.

Of the initial 22 piglets, three were euthanized due to post-surgical complications, and later two died from poor clinical conditions. The final number of piglets included in each group was *n* = 9 (SL) and *n* = 8 (WPC-PL). Birthweight was similar between WPC-PL and SL piglets, with a mean weight of 1530 g ± 235 and 1465 g ± 306, respectively. Changes in body weight in the immediate postnatal period and following period with an ileostomy were generally characterized by weight loss in both groups, albeit with lower weight loss in WPC-PL versus SL (−12.4 ± 12.3 versus −17.5 ± 14.9 g/(kg·day), *p* > 0.05). We were able to quantify stoma output reliably in a subfraction of the piglets (*n* = 5–10 piglets), see Figure 3. Based on this, we found that the fat absorption coefficient was similar for WPC-PL and SL (95.4% ± 2.5 in WPC-PL versus 95.8% ± 2.3 in SL) on day three, declining to 85.7% ± 9.4 in WPC-PL versus 83.2% ± 14.26 in SL on day seven.

Due to poor clinical conditions, three piglets did not receive their last feeding, and they were excluded. Plasma triglyceride levels one hour postprandial did not differ between groups, reaching a level in the WPC-PL piglets (*n* = 7) of 0.25 mmol/L ± 0.12 and in the SL piglets (*n* = 6) of 0.15 mmol/L ± 0.03. Organ weights and histological fat accumulation score in the proximal intestine were similar between the groups. Details are provided in Supplementary Table S1 and Figure S1.

### 4.4. In Vivo Study 2—In Vivo Fat Absorption Kinetics of Pure Emulsions

**Figure 4:**
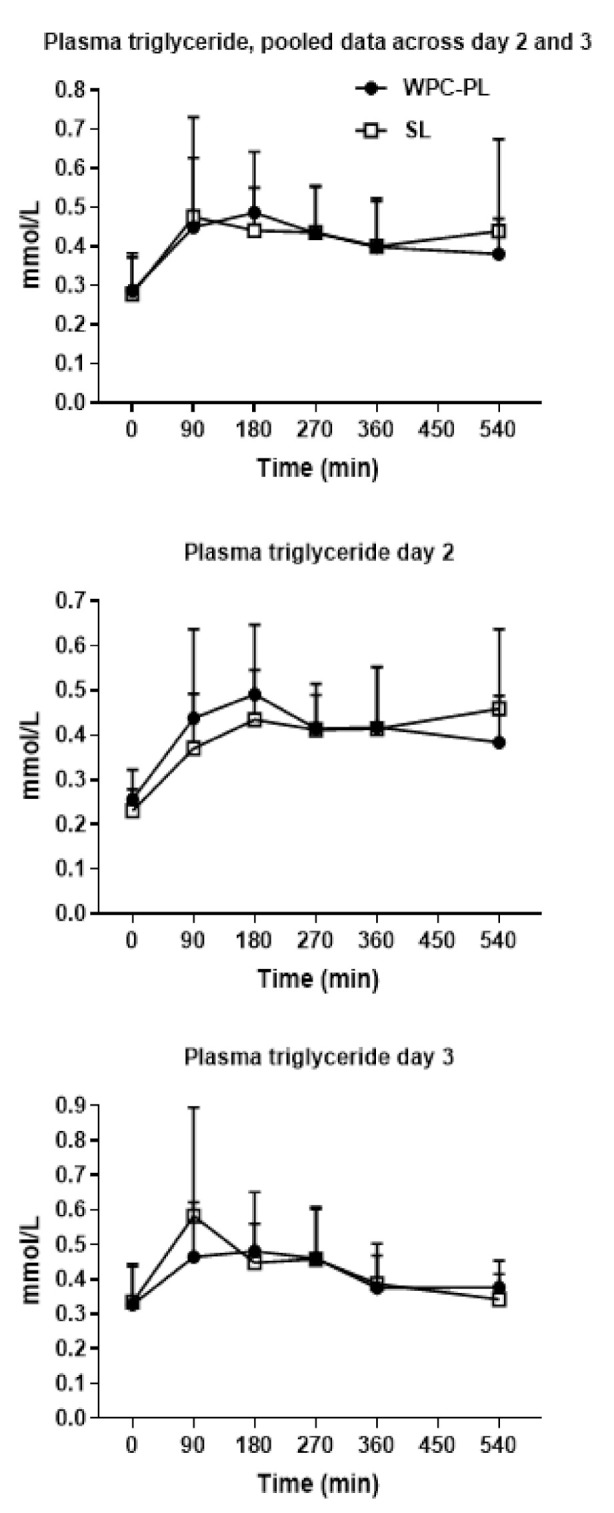
Triglyceride levels in plasma, mmol/L, as pooled data across day two and three in upper panel (WPC-PL *n* = 16, SL *n* = 18), and data from day two in middle panel (WPC-PL *n* = 9, SL = 9) and three in lower panel (WPC-PL *n* = 9, SL *n* = 7) separately. Values presented as means ± SD.

Using the cross-over design, the final group sizes were *n* = 16 for WPC-PL and *n* = 18 for SL. Mean body weight at birth was 933 g ± 277 (WPC-PL) and 969 g ± 226 (SL). Triglyceride levels in plasma were similar for WPC-PL and SL on both days two and three, and for the pooled values across both days (Figure 4).

Peak plasma triglyceride level was reached between 60–120 min after bolus, with no noticeable difference between the groups. Other plasma measurements, including cholesterol, glucose, ALAT, ASAT, and creatinine, were also similar for WPC-PL and SL. The only exception from this was the WPC-PL piglets’ baseline level of total bilirubin, which was significantly higher relative to SL, *p* = 0.03. All biochemistry data are available in Supplementary Table S2.

### 4.5. In Vivo Study 3—In Vivo Fat Absorption Kinetics from Complete Formulas

Final number of piglets were *n* = 17 (SL), *n* = 14 (WPC-PL) and *n* = 15 (WPC-A-EV). Birthweight was similar between SL (1124 g ± 264), WPC-PL (1077 g ± 225) and WPC-A-EV (1108 g ± 260) fed piglets, weight gain was similar for SL (−5.39 g/(kg·day) ± 11.6), WPC-PL (−2.19 g/(kg·day) ± 10.1) and WPC-A-EV (−1.24 g/(kg·day) ± 7.4). Three piglets were euthanized due to clinical complications unrelated to the diets.

**Figure 5:**
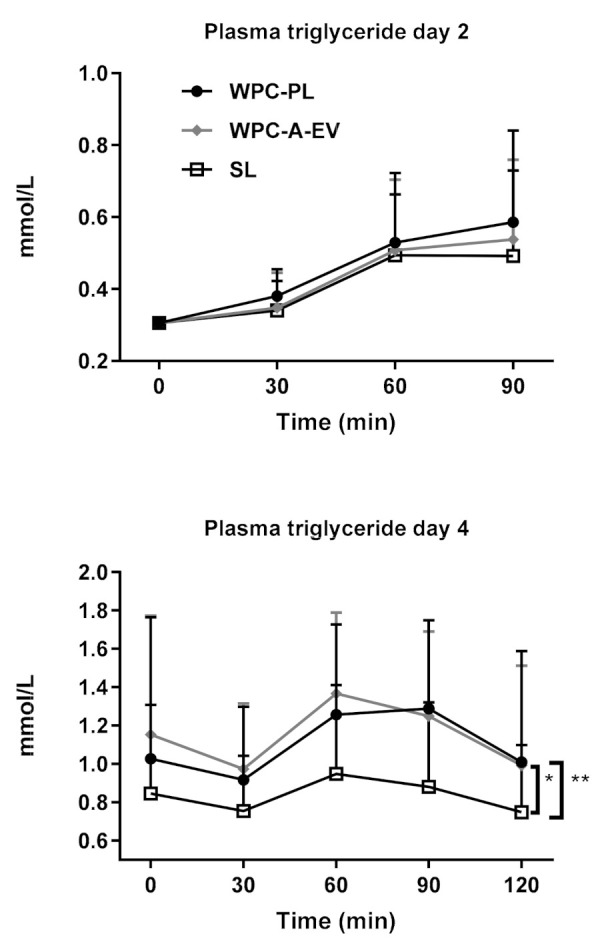
Triglyceride levels in plasma at day two (WPC-A-EV *n* = 16, WPC-PL *n* = 16, SL *n* = 17) and four (WPC-A-EV *n* = 15, WPC-PL *n* = 14, SL *n* = 17), baseline sample and 30, 60, 90 and 120 min after a test bolus was given. Presented as mean ± SD. * *p* < 0.01. ** *p* < 0.001.

Following the bolus administration on day two, plasma triglyceride levels increased similarly for all three groups over the entire 90 min sampling time relative to the common baseline level. (Figure 5, upper panel). When the bolus test was repeated on day four, plasma triglyceride level again peaked between 60–90 min, and importantly, the WPC-A-EV and WPC-PL groups had significantly higher plasma triglyceride levels compared to the SL group, *p* < 0.01 and *p* < 0.001 respectively. The values for WPC-A-EV and WPC-PL were similar (Figure 5, lower panel).

### 4.6. Gastric Fat Concentration and Lipase Activity—Related to In Vivo Study 3

**Figure 6:**
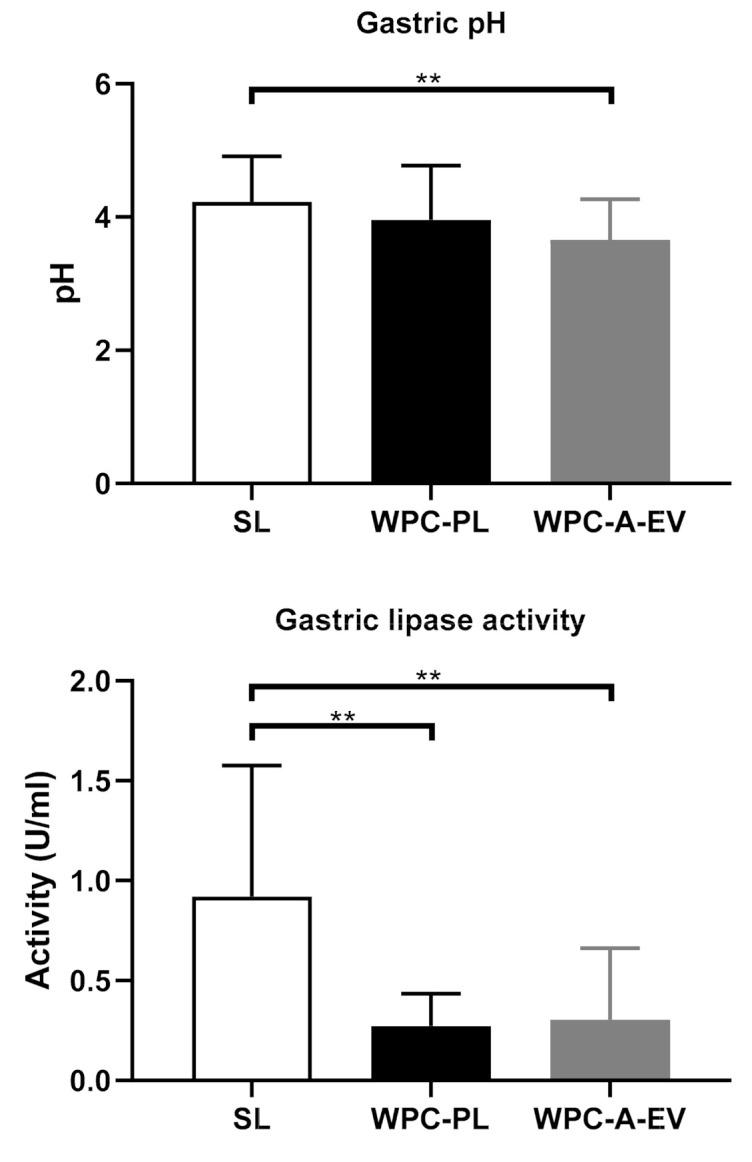
(**Top panel**): Gastric pH for WPC-A-EV (*n* = 14), WPC-PL (*n* = 11), and SL (*n* = 17) in gastric content collected post euthanasia. (**Bottom panel**): Gastric lipase activity for WPC-A-EV (*n* = 13), WPC-PL (*n* = 11), and SL (*n* = 16) in gastric content collected post euthanasia. ** *p* < 0.01.

As a result of the standardized feeding regimen before euthanasia, the gastric residuals were very similar across the groups, i.e., 15–16 g per pig (Supplementary Table S3). However, the fat concentration in the residuals was significantly higher in the SL piglets: 157 ± 27 mg/g, compared with WPC-PL: 108 ± 17 mg/g and WPC-A-EV: 103 ± 13 mg/g, *p* < 0.001 in both cases. Due to higher fat content in the SL group gastric residuals, the subsequent analysis of gastric lipase showed the highest activity in the SL group relative to WPC-PL and SL, which may partly be due to the higher availability of substrate (i.e., fat) for the lipase assay. Specific numbers were SL: 0.92 U/mL ± 0.63; WPC-PL: 0.27 U/mL ± 0.15 and WPC-A-EV: 0.30 U/mL ± 0.34 (*p* < 0.01 for WPC-PL and WPC-A-EV relative to SL). The gastric pH was similar between groups (Figure 6).

Further, circulatory levels of albumin, liver enzymes, creatinine, creatine kinase, glucose, phosphate, and urea, were largely similar across the groups (Supplementary Table S4).

## 5. Discussion

The most important finding was that piglets fed complete formula with WPC-PL and WPC-A-EV emulsifiers showed higher plasma triglyceride levels relative to the SL group when studying fat absorption kinetics in vivo. This notion was further substantiated in the in vitro lipolysis assays. MFGM surrounds the milk fat globule in human milk, and this membrane structure has its unique content of phospholipids, glycerolipids, cholesterol, and glycosylated membrane proteins. This provides physical properties allowing lipases to have high catalytic efficiency during gut luminal fat digestion [26]. In contrast, the fat fraction in infant formula is emulsified primarily by SL and milk proteins like β-lactoglobulin and caseins, which creates a different lipid-water interface compared with MFGM in human milk [29]. Therefore, we hypothesized that the utility of naturally occurring dairy emulsifiers isolated industrially from bovine whey, here referred to as WPC-PL, WPC-A-TAG, WPC-A-WP, and WPC-A-EV, has superior absorptive effects relative to SL in vitro and in vivo. These absorptive effects could potentially result in higher availability of lipids for brain development. Gastric lipase activity is particularly important in early life, and surprisingly our ex vivo analysis of gastric lipase activity in the stomach content showed the highest activity in the SL group relative to WPC-PL and WPC-A-EV. This may, however, partly be explained by a higher residue of undigested lipids in the SL group gastric residuals, leading to more substrate available for the ex vivo lipolysis assay. However, the gastric fat content was approximately 50% higher in the SL group relative to WPC-PL and WPC-A-EV. We normalized the gastric lipase activity to gastric fat content and found that it does not fully account for the higher lipase activity seen in the SL group, and we speculate that other regulatory mechanisms play a role.

An adequate supply of lipids, including LC-PUFA to the brain during early life, helps secure synaptogenesis and brain maturation and gives lasting effects on cognition [21]. Therefore, infant formulas should ensure LC-PUFA is available to the brain. Although efforts have been made to customize infant formulas to mimic human milk, including fortifying formula with LC-PUFA, the results have been inconclusive with regard to cognitive effects [20]. Fat absorption is lower in formula-fed newborn infants than babies fed mother’s milk, and assuming that the level of endogenous digestive enzyme activity is similar between breastfed and formula-fed infants, this indicates that factors that relate to the fat fraction per se, determine the level of lipolysis and absorption.

The interfacial layer of the lipid droplets affects the fat digestion in milk, and our results indicate that when the interfacial layer is composed mainly of phosphatidylcholine (i.e., the SL emulsions), the lipolysis is slower compared to lipid droplets emulsified with bovine dairy emulsifiers of more complex polar lipid and protein composition. This agrees with the findings from earlier studies suggesting that the pancreatic lipase has a higher affinity for a lipid surface covered with casein or whey proteins [37]. This notion is supported by the TEM and cryo-SEM microstructure micrographs, which indicate that the surface of WPC-PL and WPC-A-EV emulsified droplets is thin and smooth, which may favor the lipase to penetrate and reach the lipids. In contrast, in SL emulsions, the formation of liposomes may additionally reduce the lipase efficacy.

We used a neonatal preterm piglet model to determine the effects of WPC-PL, WPC-A-EV, and SL emulsifiers on the absorption of triglycerides. Appropriate animal models offer the potential of eliminating important confounders that exist in the comparison of breastfeeding versus formula feeding, and we suggest that preterm pigs with their resemblance to preterm infants in terms of gut and brain development [34] and post-surgical responses [38], is a valuable tool to determine these effects. Our initial study of triglyceride digestibility using an ileostomy model showed similar triglyceride absorption levels between the WPC-PL and SL groups. Plasma triglyceride levels measured one hour postprandial were numerically higher in the WPC-PL group, but this did not reach significance with the limited sample size. The preterm pigs were born on gestational day 113, i.e., 4–5 days before the expected termination date. It is possible that the degree of prematurity was too small to sensitize the digestive system toward reduced-fat uptake. In the two subsequent piglet studies, we chose to further sensitize the model by delivering the piglets on day 106 of gestation, i.e., 11–12 days before the expected term date. Yet, histological lipid stainings showed only numerically higher scores for WPC-PL and WPC-A-EV relative to SL. From the notion that fat uptake is indeed higher in WPC-PL and WPC-A-EV relative to SL, the apparently similar fat level in the mucosa may indicate that the transport of fat as chylomicrons into lymph vessels is an equally efficient process relative to absorption from the gut lumen, resulting in no net fat accumulation in the mucosa.

Several randomized controlled trials have examined the safety and efficacy of dairy phospholipid-enriched infant formulas. In one multicenter randomized controlled trial, they used two commercial phospholipid-rich ingredients and investigated its safety in infant formula [39]. It was proven safe to use in healthy term-born infants, with eczema being the only adverse effect reported. Likewise, Xionan and colleagues [40], found that a commercial dairy phospholipid-rich ingredient was safe and well-tolerated, and found no difference in skin effects relative to neither standard formula nor breastfeeding. In another randomized controlled trial, a standard formula was compared with an experimental formula enriched with the same dairy phospholipid-rich ingredient and showed improved cognition score in the experimental group using Bayley Scales of Infant and Toddler Development III at 12 months of age [32] and lower incidence of acute otitis media [41]. Whereas the dairy phospholipid-enriched group generally performed similar to a breastfed reference group, the breastfed infants performed better than both formula-fed groups on a verbal subscale. In the same infants, an analysis of lipidomics profile in serum/plasma and erythrocyte membranes at four, six, and twelve months of age showed significant differences between the dairy phospholipid-enriched formula and the standard formula groups. The difference in serum did, however, disappear six months after the intervention [42]. The discrepancy in the lipidomics profile was mainly accounted for by sphingomyelin, likely to be explained by the contribution from the dairy phospholipid ingredient. The provision of sphingomyelin into the blood circulation may provide the developing brain with this essential nutrient, yet the direct causality remains to be determined. In support of the notion that dairy phospholipids are important for brain development, Gurnida and colleagues showed positive effects of glycosphingolipids on hand-eye coordination, performance score, and total score measured with the Griffiths Mental Developmental Scale (GMDS) in term-born infants [43].

We conclude that the milk-derived emulsifiers WPC-PL and WPC-A-EV increase fat digestion and absorption of triglycerides relative to SL. The effects are seen in vivo when the emulsions are an integrated part of a complete diet, but not when the pigs were fed pure emulsions. Higher levels of lipid hydrolysis, as indicated in vitro, suggest that the higher absorptive rates in vivo result from increased lipase activity when WPC-PL and WPC-A-EV are used as emulsifiers relative to SL. It remains to be determined whether emulsification with these milk-derived polar lipids can affect brain development.

## Figures and Tables

**Table 1:** Emulsifier composition of protein, neutral fat, and phospholipids.

	WPC-PL	WPC-A-TAG	WPC-A-EV	WPC-A-WP	SL
Percent of total:					
Proteins	72.7	53	76	89.3	N/A
Neutral fat	17.8	41	18	1.87	N/A
Phospholipids (PL)	7.1	12.4	8.9	0.75	43.3
Percent of PL:					
PC	27.4	26	27.3	28	31.2
PE	29	29	28	28	16.6
PI	7.2	5.5	5.6	5.3	26.2
PS-Na	6	10.6	9.8	9.3	0.6
SM	30.2	26.9	27.5	24	0
Other	0.2	2	1.8	5.4	25.4

PL: phospholipids; PC: phosphatidylcholine; PE: phosphatidylethanolamine; PI: phosphatidylinositol; PS-Na: phosphatidylserine-sodium; SM: sphingomyelin, N/A: not assessed; WPC-A-EV: whey protein concentrate from acid whey-enriched in extracellular vesicles (WPC-A-EV), WPC enriched in phospholipids; WPC-A-TAG: whey protein concentrate from acid whey enriched in triglycerides; WPC-A-WP: whey protein concentrate from acid whey enriched in soluble whey protein; SL: soy lecithin.

**Table 2:** Final in vitro gastric- and intestinal digestion assay concentrations (mM). The contribution of CaCl_2_ and NaCl from the emulsions is included in the shown concentrations.

	Final Assay Composition	
Compound ^1^	Simulated Gastric Digestion, mM	Simulated Intestinal Digestion, mM
NaCl	10.4	51.8
Tris	2.0	2.2
Maleic acid	2.0	2.2
CaCl_2_	10.1	5.9
Sodium taurocholate	0.0	0.5
Phospholipid	0.0	0.1

^1^ NaCl was purchased from VWR (Darmstadt, Germany), Tris from ICN Biomedicals (Santa Ana, CA, USA), phospholipids (phosphatidylcholine) from Lipoid (Köln, Germany), maleic acid, and sodium taurocholate from Sigma Aldrich.

## Data Availability

All generated data are stored on pass word protected servers at University of Copenhagen, and can be made available upon request.

## Supplementary Materials

The following are available online at https://www.mdpi.com/2072-6643/13/2/410/s1, Table S1. Organ weight of pigs equipped with an ileostomy and fed milk diets with SL or WPC-PL for seven days; Table S2. Circulatory markers in pigs with boluses of pure emulsion; Table S3. Organ weight in pigs fed complete formulas with either SL, WPC-PL, or WPC-A-EV; Table S4. Circulatory markers in pigs fed complete formulas with either SL, WPC-PL, or WPC-A-EV as an emulsifier; Figure S1. Higher score indicates higher fat infiltration, as assessed subjectively by several independent observers who were blinded to the treatment groups [44].
