# Supplementary material for: Bovine Milk-Derived Emulsifiers Increase Triglyceride Absorption in Newborn Formula-Fed Pigs
Source: Nutrients. 2021 Jan 28;13(2):410. doi: 10.3390/nu13020410 (PMC7912295; doi:10.3390/nu13020410)
Supplement: Supplementary file 1 [file nutrients-13-00410-s001.pdf]

Supplementary material:

Methods for In Vivo Digestibility Pilot Study

Within four hours of birth, the piglets were fitted with a vascular catheter (4F; Portex, Kent, UK), inserted into the dorsal aorta via the transected umbilical cord, and used for parenteral nutrition support (PN). Further, the piglets were fitted with an orogastric feeding tube (8F Portex), inserted via the cheek for enteral nutrition (EN) [44]. Both catheters were sutured to the skin. To provide passive immunity, maternal plasma, collected at the time of cesarean section, was infused via the umbilical catheter at a volume of 25 mL/kg body weight within the first 24 h after birth. Parenteral nutrition with amino acids and glucose, but not fat, was initiated quickly after birth using a modified composition of Kabiven and Vamin (both Fresenius, Bad Homburg, Germany), to meet the nutritional requirements of the piglets. PN was infused continuously via the umbilical catheter at 6 mL/(kg·h) the first 48 h decreasing to 3 mL/(kg·h) on the last study day. If a piglet lost its vascular catheter during the study, PN was replaced with enteral supplementation of fluid and electrolytes (Revolyt, Gunnar Kjems, Copenhagen, Denmark). Approximately 24–36 h after birth, all pigs were anesthetized and prepared for abdominal surgery, as described previously. The small intestine was transected at the ileocecal junction and exteriorized in the left flank region as a stoma. Stoma bags were glued to the skin and emptied as needed.

Establishment of Ileostomy

To determine the level of lipid absorption between the experimental diets, the piglets were equipped with an ileostomy for the quantitative collection of stoma output. The piglets were prepared for surgery 24 h after birth using pre-medication with an intramuscular injection of a mixture of ketamin (5 mg/kg, Ketaminol Vet, 100 mg/mL, MSD Animal Health, Copenhagen, Denmark) and midazolam (0.25 mg/kg, Midazolam Accord, 1 mg/mL, Accord Healthcare Limited, North Harrow, UK). The piglets were anesthetized with inhalation of sevoflurane (SevoFlo, Orion, Copenhagen, Denmark) via a mask, and Fthen intubated to maintain anesthesia and ventilation with a Hallowell anesthesia workstation (Anesthesia workstation w/100 mL, Hallowell EMC, Pittsfield, MA, USA). They were placed in a supine position and kept warm with heated rice pads and plastic covers. The abdominal skin was cleaned, sterilized, and covered with a Foliodrape (Hartmann, Avondale, New Zealand), and local analgesia (Xylocain 2%, astraZenica, Macclesfield, UK) was injected. A midline 5 cm caudal incision was performed, and the ilium was identified and transected about five cm from the ileocecal valve. The proximal end of the transected ilium was passed through an incision in the left abdominal wall and sutured to the skin with Monocryl 4.0 (Ethicon, Johnson & Johnson, NJ, USA). The distal end of the transected ilium was closed with a purse-string suture Monocryl 4.0 (Ethicon, NJ, USA), and left intra-abdominally. The skin around the stoma was carefully shaved, and a stoma-bag (Coloplast A/S, Humlebaek, Denmark) with adhesive glue was applied to the skin. Following the surgical procedure, the piglets were placed in their respective incubators and provided with extra oxygen before and after extubation, until ventilation was normalized.

Table S1. Organ weight of pigs equipped with an ileostomy and fed milk diets with SL or WPC-PL for seven days.

Organ Weights [1]	SL	WPC-PL	Diet Effects
Heart (g/kg)	7.35 ± 1.47	6.37 ± 1.04	p = 0.09
Lungs (g/kg)	20.9 ± 6.55	22.8 ± 9.06	ns
Liver (g/kg)	28.8 ± 7.18	24.0 ± 2.62	ns
Kidney (g/kg)	8.67 ± 2.05	7.72 ± 0.97	ns
Spleen (g/kg)	2.10 ± 0.74	1.80 ± 0.47	ns
Adrenal glands (g/kg)	0.34 ± 0.10	0.29 ± 0.09	ns
Proximal part of small intestine (g/kg)	9.30 ± 2.48	10.4 ± 1.55	ns
Mid part of small intestine (g/kg)	9.39 ± 1.63	10.2 ± 1.99	ns
Distal part of small intestine (g/kg)	10.7 ± 2.19	11.1 ± 2.23	ns
Empty stomach (g/kg)	8.66 ± 1.77	8.75 ± 1.64	ns
Length of small intestine (cm)	341 ± 52	389 ± 48	p = 0.03
Brain region weights [2]			
Total brain weight[1] (g/kg)	24.5 ± 5.88	23.2 ± 4.87	ns
Cerebrum (g/g)	0.80 ± 0.01	0.80 ± 0.01	ns
Cerebellum (g/g)	0.11 ± 0.01	0.11 ± 0.01	ns
Brainstem (g/g)	0.09 ± 0.01	0.10 ± 0.01	ns
Hippocampus (g/g)	0.02 ± 0.002	0.02 ± 0.003	ns
Striatum (g/g)	0.01 ± 0.001	0.01 ± 0.01	ns
Remnant (g/g)	0.34 ± 0.01	0.34 ± 0.02	ns
Water in the brain (%)	83.5 ± 0.24	83.3 ± 0.42	ns

ns = not significant. [1] Relative to total body weight, [2] Relative to total brain weight. Values are mean ± SD.

Table S2**.** Circulatory markers in pigs with boluses of pure emulsion.

Biochemistry [1]	SL	WPC-PL	Diet Effects
Albumin (g/L)	6.73 ± 0.72	6.17 ± 0.87	ns
Total protein (g/L)	19.1 ± 1.3	19.0 ± 1.54	ns
ASAT (U/L)	15.1 ± 2.69	14.2 ± 2.46	ns
ALAT (U/L)	9.35 ± 1.46	9.62 ± 1.89	ns
Total bilirubin (mol/L)	0.24 ± 0.44	0.69 ± 1.01	ns [2]
Gamma-glutamyl transferase (U/L)	15.1 ± 6.56	14.8 ± 5.98	ns
Creatinine (μmol/L)	63.4 ± 9.90	61.9 ± 7.68	ns
Creatinine kinase (U/L)	79.5 ± 71.8	70.2 ± 37.11	ns
Inorganic Phosphate (mmol/L)	1.74 ± 0.9	1.74 ± 0.21	ns
Blood urea nitrogen (mmol/L)	4.04 ± 0.87	4.01 ± 0.74	ns
Cholesterol (mmol/L)	1.21 ± 0.17	1.19 ± 0.23	ns
Glucose (mmol/L)	7.50 ± 3.11	7.56 ± 3.56	ns

ns = not significant. [1] Baseline level measured before meal bolus, [2] Background levels were significantly higher in WPC-PL piglets, p = 0.03, however, no effects of the diet were present**.** Values presented as mean ± SD.

Table S3. Organ weight in pigs fed complete formulas with either SL, WPC-PL, or WPC-A-EV.

Organ Weights [1]	SL	WPC-PL	WPC-A-EV	Diet Effects
Heart (g/kg)	7.50 ± 1.11	7.32 ± 1.13	7.38 ± 0.89	ns
Lungs (g/kg)	21.5 ± 4.13	20.9 ± 4.15	19.9 ± 3.36	ns

Liver (g/kg)	24.4 ± 3.79	25.0 ± 3.52	24.3 ± 2.23	ns
Kidney (g/kg)	8.64 ± 1.56	8.63 ± 1.39	8.07 ± 0.79	ns
Spleen (g/kg)	2.71 ± 3.68	1.60 ± 0.37	1.83 ± 0.39	ns
Full stomach [2] (g)	22.3 ± 8.03	21.8 ± 9.14	22.4 ± 9.58	ns
Empty stomach [2] (g)	6.45 ± 1.93	6.61 ± 2.77	6.63 ± 2.11	ns
Stomach content [2] (g)	15.8 ± 7.32	15.2 ± 7.56	15.8 ± 8.13	ns
Prox small intestine (g/kg)	9.11 ± 1.18	8.76 ± 1.64	9.24 ± 1.22	ns
Middle small intestine (g/kg)	8.81 ± 0.95	8.50 ± 1.21	9.24 ± 1.47	ns
Distal small intestine (g/kg)	8.80 ± 0.94	8.79 ± 1.01	8.96 ± 1.47	ns
Full colon weight (g/kg)	13.0 ± 3.05	10.8 ± 2.39	10.5 ± 2.86	p = 0.051 [4]
Length small intestine (cm)	323 ± 39	304 ± 37	314 ± 45	ns
Mean brain weights [3]				
Total brain weight [1] (g/kg)	280 ± 6.01	28.7 ± 8.37	27.9 ± 7.64	ns
Cerebrum (g/g)	0.82 ± 0.002	0.82 ± 0.002	0.82 ± 0.002	ns
Cerebellum (g/g)	0.09 ± 0.01	0.10 ± 0.01	0.10 ± 0.01	ns
Brainstem (mg/g)	88.8 ± 5.4	89.4 ± 6.30	89.7 ± 4.80	ns
Hippocampus (mg/g)	17.2 ± 2.90	17.8 ± 3.2	17.2 ± 2.90	ns
Striatum (mg/g)	8.40 ± 8.40	13.0 ± 4.20	12.2 ± 2.70	ns
Remnant (mg/g)	349 ± 9.20	340 ± 8.20	344 ± 9.60	p = 0.005 [5]
Water in the brain (%)	85.1 ± 1.44	84.7 ± 0.44	84.6 ± 0.31	ns

ns = not significant. [1] Relative to total body weight, [2] Absolute organ weight, [3] Relative to total brain weight, [4] SL compared with WPC-A-EV, [5] SL compared with WPC-PL, Values are mean ± SD.

Table S4. Circulatory markers in pigs fed complete formulas with either SL, WPC-PL, or WPC-A-EV as an emulsifier.

**Biochemistry [1]**	**Day 2**			**Day 4**			**Diet Effects**
	**SL**	**WPC-PL**	**WPC-A-EV**	**SL**	**WPC-PL**	**WPC-A-EV**	
**Albumin (g/L)**	8.20 ± 1.01	7.42± 0.92	7.82 ± 0.66	11.6 ± 0.98	11.3 ± 1.15	12.1 ± 1.43	ns [2]
Total protein (g/L)	22.4 ± 1.96	20.7± 1.61	21.6 ± 1.47	27.6 ± 1.72	27.2 ± 1.89	28.8 ± 2.90	ns [2]
ALT (U/L)	124 ± 147	136 ± 174	114 ± 131	375 ± 200	330 ± 330	280 ± 170	ns
ALAT (U/L)	12.2 ± 4.19	11.4± 2.90	11.0 ± 2.19	15.5 ± 2.98	14.5 ± 2.28	14.9 ± 1.92	ns
ASAT (U/L)	15.4 ± 4.47	17.9± 15.8	15.6 ± 5.02	17.5 ± 10.9	14.9 ± 1.96	17.3 ± 6.96	ns
Total bilirubin (µmol/L)	0.18 ± 0.39	0.06± 0.25	0.44 ± 0.81	1.59 ± 1.33	1.50 ± 1.29	1.87 ± 1.13	ns
GGT (U/L)	14.2 ± 4.75	20.2± 6.80	16.4 ± 7.26	15.4 ± 4.72	22.6 ± 6.16	18.0 ± 7.49	ns [3]
Creatinine (µmol/L)	78.1 ± 10.7	81.9± 15.1	77.9 ± 12.2	65.3± 12.4	65.4 ± 16.6	63.9 ± 9.84	ns
Creatinine kinase (U/L)	171 ± 125	164 ± 63.7	163 ± 108	40.4 ± 12.8	46.6 ± 30.2	57.9 ± 47.8	ns
Phosphate (mmol/L)	1.94 ± 0.25	1.85± 0.15	1.78 ± 0.27	1.69 ± 0.12	1.50 ± 0.15	1.59 ± 0.16	ns[4]
BUN (mmol/L)	5.51 ± 1.85	5.04± 1.55	5.00 ± 1.81	6.62 ± 1.90	5.49 ± 2.25	5.84 ± 1.30	ns
Cholesterol (mmol/L)	1.46 ± 0.38	1.34± 0.25	1.46 ± 0.27	2.53 ± 0.38	2.62 ± 0.52	2.89 ± 0.70	ns

Glucose (mmol/L)	6.38 ± 6.38	7.82± 7.82	5.59 ± 2.14	5.70 ± 3.11	5.76 ± 2.74	4.79 ± 2.72	ns

ns = not significant, [1] Baseline level measured before meal bolus, [2] WPC-PL had significantly higher background level compared with SL on day two, however, no effects of the diet were present, [3] WPC-PL had significantly higher background level compared with SL on both days, however, no effects of the diet were present, [4] SL had significantly higher background level compared with WPC-PL on day four, however, no effects of the diet were present. Values presented as mean ± SD.

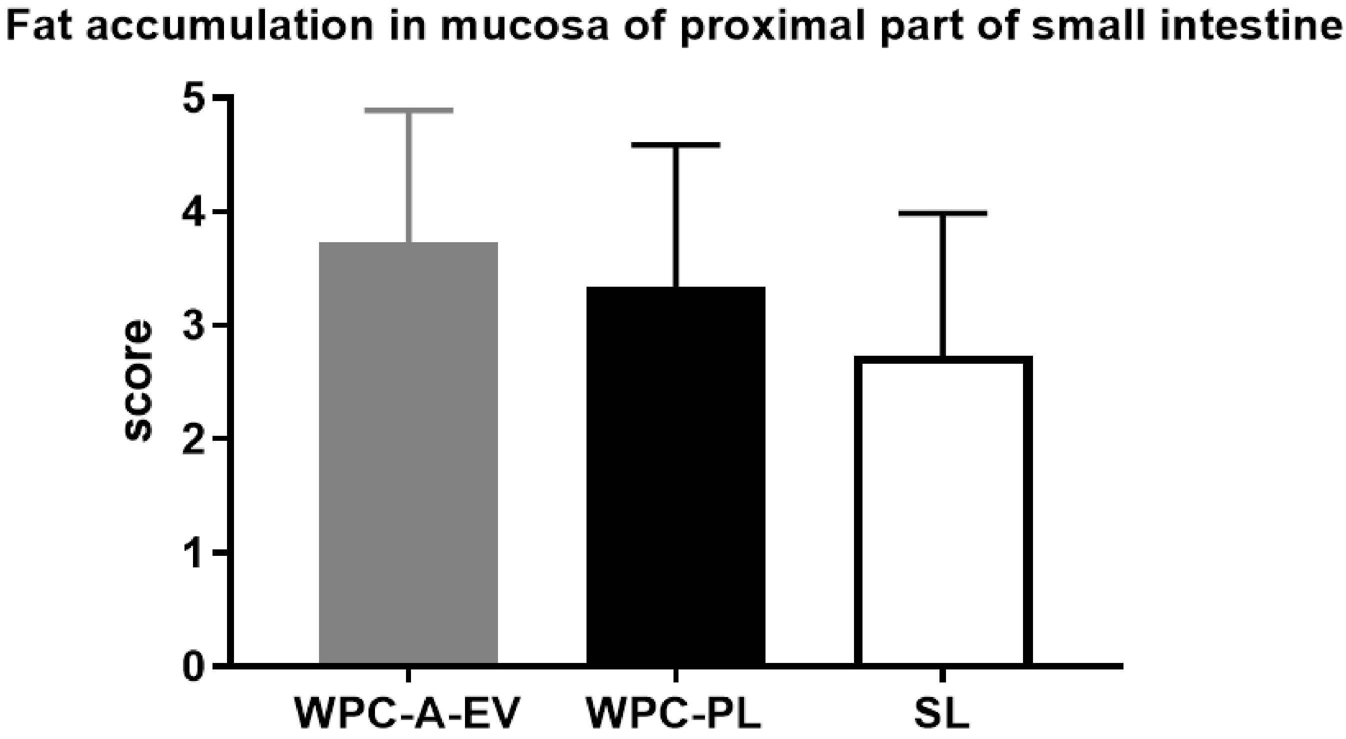

Figure S1. Higher score indicates higher fat infiltration, as assessed subjectively by several independent observers who were blinded to the treatment groups.
